# Endoscopic ultrasound-guided radiofrequency ablation in the treatment of pancreatic ductal adenocarcinoma: when science tries to increases hope

**DOI:** 10.1590/0102-672020260000032e1961

**Published:** 2026-08-17

**Authors:** José Celso Ardengh, Rafael Kemp, José Sebastião dos Santos

**Affiliations:** 1 Hospital Moriah, Digestive Endoscopy – São Paulo (SP), Brazil.; 2 Universidade de São Paulo, Faculty of Medicine Ribeirão Preto, Hospital de Clínicas, Department of Surgery and Anatomy – Ribeirao Preto (SP), Brazil.; 3 Universidade Federal de São Paulo, Imaging Diagnostic – São Paulo (SP), Brazil.

**Keywords:** Pancreatic Neoplasms, Endoscopic Ultrasound-Guided Fine Needle Aspiration, Radiofrequency Ablation, Palliative Care, Neoplasias Pancreáticas, Aspiração por Agulha Fina Guiada por Ultrassom Endoscópico, Ablação por Radiofrequência, Cuidados Paliativos

## Abstract

Pancreatic ductal adenocarcinoma (PDAC) is the most common form of pancreatic cancer and remains the most lethal malignancy of the digestive system. Despite recent advances, surgical treatment remains the only potentially curative option. Most patients are diagnosed with locally advanced or disseminated disease, and chemotherapy is the only indicated treatment. Pancreatic resection rates in centers that do not perform vascular resection are around 15–20% of diagnosed cases, while in specialized centers with vascular expertise, they may reach 30–45%. Ablative therapies have been investigated as local alternatives for unresectable tumors. Radiofrequency ablation (RFA) has proven efficacy in the treatment of hepatic neoplasms, and has recently been explored in PDAC. Endoscopic ultrasound-guided radiofrequency ablation (EUS-RFA) represents a minimally invasive alternative for thermal ablation of pancreatic tumors. Early experience suggests that may serve as a palliative modality for pain or obstruction control, and to facilitate chemotherapy. However, this treatment is considered experimental and requires procedural standardization. Careful patient selection is essential, prioritizing cases without metastatic spread, with tumors accessible by EUS and without extensive vascular invasion.

## ARTICLE HIGHLIGHTS

- Endoscopic ultrasound-guided radiofrequency ablation should not replace surgery in resectable pancreatic ductal adenocarcinoma.
- The endoscopic ultrasound-guided radiofrequency ablation should be considered as a palliative option within multidisciplinary protocols.
- Endoscopic ultrasound-guided radiofrequency ablation is an isolated palliative modality, and considered an option to treat the pancreatic ductal adenocarcinoma.

## RESUMO

O adenocarcinoma ductal pancreático (ADP) é a forma mais comum de câncer pancreático e continua sendo a neoplasia maligna mais letal do sistema digestivo. Apesar dos avanços recentes, o tratamento cirúrgico permanece a única opção potencialmente curativa. A maioria dos pacientes é diagnosticada com doença localmente avançada ou disseminada, sendo a quimioterapia o único tratamento indicado. As taxas de ressecção pancreática em centros que não realizam ressecção vascular giram em torno de 15–20% dos casos diagnosticados, enquanto em centros especializados com *expertise* vascular podem chegar a 30–45%. Terapias ablativas têm sido investigadas como alternativas locais para tumores irressecáveis. A ablação por radiofrequência (ARF) demonstrou eficácia no tratamento de neoplasias hepáticas e foi recentemente explorada no adenocarcinoma pancreático avançado. A ablação por radiofrequência guiada por ultrassonografia endoscópica representa uma alternativa minimamente invasiva à ablação térmica de tumores pancreáticos. A experiência inicial sugere que pode servir como modalidade paliativa para o controle da dor ou obstrução, bem como para facilitar a quimioterapia. No entanto, é um tratamento ainda considerado experimental e requer padronização do procedimento. A seleção criteriosa dos pacientes é essencial, priorizando casos sem disseminação metastática, com tumores acessíveis por ultrassonografia endoscópica e sem extensa invasão vascular.

## INTRODUCTION

Pancreatic ductal adenocarcinoma (PDAC) is the most common form of pancreatic cancer, and remains the most lethal malignancy of the digestive system, despite advances in understanding its natural history, diagnostic tools, and progress in genomics and proteomics^1,5,7^. Surgical treatment remains the only potentially curative option. However, most patients are diagnosed with locally advanced or disseminated disease, and remain so even after neoadjuvant chemotherapy^8,10^.

Pancreatic resection rates for PDAC in centers that do not perform vascular resection are around 15–20% of diagnosed cases, while in specialized centers with vascular expertise, they may reach 30–45% for initially borderline or locally advanced cases after neoadjuvant chemotherapy. Major postoperative complication rates range from 42 to 47%, with a 90-day mortality of 7–10%, which remains high^2,8,10,12^.

In this context, ablative therapies have been investigated as local alternatives for unresectable tumors. Radiofrequency ablation (RFA) has proven efficacy in the treatment of hepatic neoplasms, and has recently been explored in PDAC^1,10^. Although percutaneous RFA is technically feasible, its use is limited by anatomical challenges, risk of complications, and radiation exposure^8^.

Endoscopic ultrasound-guided radiofrequency ablation (EUS-RFA) represents a minimally invasive alternative for thermal ablation of pancreatic tumors, including unresectable PDAC, resectable PDAC in patients unfit for surgery, cystic neoplasms, and neuroendocrine tumors. Early experience suggests that EUS-RFA may serve as a palliative modality for pain or obstruction control, to facilitate neoadjuvant chemotherapy (nQT), or as a potential method for tumor downstaging, improving the likelihood of surgical resection in selected cases^3,13,15^. However, EUS-RFA is still considered experimental, and requires procedural standardization. Careful patient selection is essential, prioritizing cases without metastatic spread, with tumors accessible by EUS and without extensive vascular invasion.

Observational studies report technical success rates between 70 and 100%, with modest tumor mass reduction — slightly above 50% in most cases — and acceptable safety in experienced centers. However, most available data are from observational studies, case series, or small cohorts^6^. Future randomized clinical trials are needed to provide definitive evidence regarding usability, limitations, risks, and outcomes. Relevant aspects include defining optimal power, duration, number of sessions, operator experience, adverse event risk (pancreatitis, duodenal/hepatic abscess, biliary or duodenal stricture), biomarker use, advanced imaging (3 Tesla Magnetic Resonance Imaging — 3T MRI; Positron Emission Tomography/Computed Tomography — PET/CT) for validation, immunological effects, and above all, its still inconclusive impact on survival^3,4,9,11,14^.

EUS-RFA should be performed in reference centers, preferably under clinical protocols or prospective studies, with detailed informed consent. Outside this context, EUS-RFA should not replace surgery in resectable PDAC, and should be considered as a palliative option within multidisciplinary protocols. The use of EUS-RFA as an isolated palliative modality may be considered individually, particularly in patients with contraindications to surgery or radiotherapy^9,11^.

## Data Availability

The datasets generated and/or analyzed during the current study are available from the corresponding author upon reasonable request.
